# Efficacy of Injectable Platelet-Rich Fibrin (I-PRF) in Managing Temporomandibular Joint Pain: A Prospective Clinical Study

**DOI:** 10.7759/cureus.54367

**Published:** 2024-02-17

**Authors:** Nithin Kumar, Mariea Francis, Sai Sindhu VK, Varun Ramachandra, P Vijay Anilkumar, Mohammad Fahad Khan

**Affiliations:** 1 Department of Oral and Maxillofacial Surgery, School of Dentistry, Central Asian University, Tashkent, UZB; 2 Department of Oral and Maxillofacial Surgery, Sankalchand Patel University, Visnagar, IND; 3 Department of Oral and Maxillofacial Surgery, RVS Dental College and Hospital, Coimbatore, IND; 4 Department of Oral and Maxillofacial Surgery, Manubhai Patel Dental College, Vadodara, IND; 5 Department of Oral and Maxillofacial Surgery, GITAM Dental College and Hospital, Visakhapatnam, IND; 6 Department of Oral and Maxillofacial Surgery, Chandra Dental College and Hospital, Barabanki, IND

**Keywords:** patient-reported outcomes, quality of life, pain management, injectable, platelet-rich fibrin, temporomandibular joint

## Abstract

Background: Temporomandibular joint (TMJ) pain is a common condition that can significantly impact an individual's quality of life. Current treatment options often fall short of providing long-lasting relief. So, this prospective clinical study aimed to investigate the efficacy of injectable platelet-rich fibrin (I-PRF) in mitigating TMJ pain.

Methods: A total of 68 participants, aged 18-60 years, clinically diagnosed with TMJ pain, were recruited from dental clinics and specialist referrals. Participants were randomly assigned to either the intervention group (I-PRF injections) or the control group (placebo). Primary outcome measures included TMJ pain intensity and jaw function, assessed using the Visual Analog Scale and functional examinations, respectively. Secondary outcomes comprised patient-reported outcomes (PROs) on quality of life and satisfaction. Data were collected at baseline and six, 12, and 24 weeks post intervention.

Results: Baseline characteristics demonstrated successful randomization, with no significant differences in age, gender, or TMJ pain duration between groups. Post intervention, the intervention group exhibited a significant and sustained reduction in TMJ pain intensity compared to controls (p<0.001). Improvements in jaw function were also notable in the intervention group at all follow-up time points (p<0.001). PROs related to quality of life and satisfaction substantially increased in the intervention group compared to controls (p<0.001).

Conclusion: I-PRF demonstrated significant efficacy in reducing TMJ pain intensity, improving jaw function, and enhancing PROs. These findings support the consideration of I-PRF as a valuable therapeutic intervention for individuals with TMJ pain.

## Introduction

Temporomandibular joint (TMJ) pain is a prevalent and multifaceted condition that significantly affects the quality of life for a considerable number of individuals worldwide [1]. It encompasses a spectrum of disorders involving the TMJ, masticatory muscles, and associated structures, often manifesting as pain, restricted jaw movement, and functional limitations [2]. The etiology of TMJ pain is complex, involving factors such as trauma, parafunctional habits, psychosocial stress, and inflammatory processes [3]. Conventional treatments for TMJ pain include pharmacotherapy, physical therapy, occlusal splints, and stress management [4]. However, these approaches may have limitations in addressing the diverse underlying causes and providing sustained relief. In recent years, regenerative medicine has gained attention as a potential avenue for managing TMJ disorders [5]. Injectable platelet-rich fibrin (I-PRF), a form of autologous platelet concentrate, has demonstrated promising results in promoting tissue regeneration and modulating inflammatory processes [6].

Platelet-rich fibrin (PRF) is derived from the patient's own blood, containing a concentrated mixture of platelets, leukocytes, and growth factors [7]. While PRF has shown success in various medical and dental applications, the injectable form (I-PRF) presents a novel approach for targeted delivery, potentially enhancing its therapeutic effects [8]. However, despite growing interest in the application of I-PRF, there remains a need for rigorous clinical investigations to assess its efficacy in managing TMJ pain. This study aims to contribute to the existing knowledge by conducting a prospective clinical study. The primary objective is to evaluate the efficacy of I-PRF injections in mitigating TMJ pain and improving jaw function in individuals clinically diagnosed with TMJ pain. Through the utilization of validated outcome measures, including pain intensity assessments and functional examinations, this research seeks to provide comprehensive insights into the potential benefits of I-PRF for TMJ disorders.

In light of the complex nature of TMJ pain and the need for diversified treatment modalities, this study endeavors to explore the therapeutic potential of I-PRF as a regenerative intervention. The outcomes of this investigation may contribute to the development of evidence-based approaches for managing TMJ pain, addressing a critical gap in the current therapeutic landscape.

## Materials and methods

Study design and participants

Inclusion criteria had participants aged 18-60 years, clinically diagnosed with TMJ pain based on predetermined criteria, recruited from dental clinics and specialist referrals after providing informed consent. Exclusion criteria included a history of allergic reactions to blood products, pregnancy or lactation, severe systemic diseases affecting TMJ health, recent TMJ surgery, or injections within the last six months, and contraindications to injections. Randomization was carried out using computer-generated random numbers, with participants evenly assigned to either the intervention group receiving I-PRF injections or the control group receiving a placebo or standard treatment. The study maintained a double-blind design with an ethical letter from the Institutional Review Board Committee of Central Asian University (approval number: IEC/CAU/SD/2021/22).

Preparation of I-PRF

For the preparation of I-PRF, a device centrifuge (Hettich® EBA 20 centrifuge, Sigma-Aldrich, St. Louis, MO) was employed to process 20 mL of peripheral blood obtained from the ulnar vein, collected in sterile vacuum tubes for single use [9]. These tubes underwent centrifugation at 700 rpm for three minutes, resulting in platelet concentrates in liquid form rather than clots. This liquid, rich in white blood cells, platelets, and growth factors, possesses enhanced healing properties [10].

Injection technique procedure

In terms of the injection technique using a medical syringe, the oral surgeon initiated the procedure by disinfecting the TMJ surgical site with povidone. The needle entry point was determined based on anatomical features, following the approach suggested by McCain and Hossameldin [11]. The procedure began with establishing a line from the middle of the tragus to the lateral angle of the eye on the same side. The injection needle was introduced approximately 10 mm from the middle of the tragus and about 2 mm down the previously marked line. An insulin syringe (27-gauge in diameter, approximately 1 cm in length) was used, entering wholly. The injection continued until the patient sensed pressure, and the plunger retracted upon removing the finger, after which the needle was immediately withdrawn. No other forms of TMJ disorder treatment were administered during the study period. The injection protocol involved each patient receiving two injections of 1 mL each, spaced one week apart. After the first injection, patients underwent examination, results were recorded, and the second injection was administered in the same joint using the same method. Following the second injection, patients were monitored for 24 months, marking the conclusion of the study [9-11].

Control group (no-treatment control)

In the control group, participants did not receive any active treatment; instead, they were designated as a no-treatment or waitlist control. This design allowed for a comparison between the effects of I-PRF in the intervention group and the natural course of the condition in the control group. The no-treatment approach helps assess the specific therapeutic impact of I-PRF by controlling for non-specific factors like the placebo effect. While the control group did not receive an active placebo, this design choice provides valuable insights into the relative efficacy of I-PRF compared to the absence of intervention. The study maintained its double-blind structure to minimize biases and enhance the reliability of findings.

Outcome measures

The primary focus of this outcome measure is to comprehensively evaluate TMJ pain intensity experienced by participants throughout the study. The assessment was conducted using a validated pain scale, specifically the Visual Analog Scale (VAS), renowned for its reliability and sensitivity in capturing subjective pain experiences. Participants were required to mark their perceived level of TMJ pain on a 10-cm line, anchored by "no pain" at one end and "worst possible pain" at the other. This process was administered at multiple time points, including baseline and specified follow-up intervals, to capture the dynamic nature of pain experiences throughout the study.

Jaw function, a pivotal aspect of TMJ health, was thoroughly assessed through a series of functional examinations. These assessments provided a nuanced understanding of the impact of I-PRF on the participant's ability to perform essential jaw movements. Specifically, maximal mouth opening, a key parameter indicative of TMJ mobility and flexibility, was measured using standardized techniques. Additionally, lateral excursions, which involve side-to-side movements of the jaw, were meticulously evaluated. These functional assessments were conducted at baseline to establish a reference point and at specified follow-up time points to monitor any changes in jaw function throughout the intervention period.

A comprehensive assessment of patient-reported outcomes (PROs) related to quality of life and satisfaction was undertaken using carefully selected and validated questionnaires. Participants in both the intervention and control groups were asked to provide subjective feedback on various aspects, including pain impact on daily activities, emotional well-being, and overall satisfaction with the treatment received. The designated questionnaires encompassed well-established instruments, the Short Form Health Survey (SF-36) for quality of life and the Patient Satisfaction Questionnaire, tailored to capture relevant dimensions specific to TMJ pain.

Statistical analysis

Statistical analysis utilized descriptive statistics for summarizing baseline characteristics and appropriate statistical tests (e.g., t-tests, ANOVA) for analyzing between-group differences in primary and secondary outcomes. In the statistical analysis, measurements were assessed for statistical significance using a predefined alpha level (significance level) of 0.05 (α=0.05). The data were presented descriptively, with continuous variables shown as means±standard deviations. A p-value of less than 0.05 (p<0.05) was considered statistically significant. 

## Results

The baseline characteristics table reveals a well-randomized distribution of participants between the intervention and control groups, as evidenced by the non-significant differences in mean age (p=0.62), gender distribution (p=0.81), and duration of TMJ pain (p=0.47) (Table 1).

**Table 1:** Baseline characteristics of the participants TMJ: temporomandibular joint

Characteristic	Intervention group (n=34)	Control group (n=34)	P-value
Age (years), mean±SD	42.5±8.2	41.8±7.5	0.62
Female, n (%)	25 (73.5)	26 (76.5)	0.81
Duration of TMJ pain (months)	18.4±6.7	19.2±7.1	0.47

This successful randomization ensures that any observed differences in outcomes can be more confidently attributed to the intervention rather than pre-existing variations in participant characteristics. Moving to the primary outcome measures, the TMJ pain intensity table demonstrates that, at baseline, there is no significant disparity between the intervention and control groups (p=0.42). However, post-intervention assessments at six, 12, and 24 weeks reveal a compelling pattern. The intervention group experiences a noteworthy and statistically significant reduction in TMJ pain intensity compared to the control group (p<0.001) (Table 2).

**Table 2:** TMJ pain intensity TMJ: temporomandibular joint

Time point	Intervention group (n=34)	Control group (n=34)	P-value
Baseline	6.2±1.5	6.0±1.4	0.42
6 weeks post intervention	3.2±1.2	5.8±1.3	<0.001
12 weeks post intervention	2.5±1.0	5.6±1.2	<0.001
24 weeks post intervention	2.0±0.8	5.4±1.1	<0.001

This sustained effect over the follow-up period suggests a meaningful therapeutic impact of I-PRF in mitigating TMJ pain. Analyzing the jaw function outcomes, baseline measurements show no significant differences between the intervention and control groups (p=0.68). Subsequent assessments at six, 12, and 24 weeks post intervention, however, demonstrate a substantial improvement in jaw function within the intervention group compared to controls (p<0.001) (Table 3).

**Table 3:** Jaw function

Time point	Intervention group (n=34)	Control group (n=34)	P-value
Baseline	32.1±5.7	31.8±5.5	0.68
6 weeks post intervention	40.5±4.8	31.2±5.2	<0.001
12 weeks post intervention	42.2±4.1	30.8±4.9	<0.001
24 weeks post intervention	43.8±3.5	30.5±4.5	<0.001

This finding suggests that beyond pain relief, I-PRF may positively influence the functional aspects of the TMJ. Turning to the secondary outcome measures, the PROs table indicates that baseline scores for quality of life and satisfaction are comparable between the two groups (p=0.82). Post-intervention assessments at six, 12, and 24 weeks reveal a remarkable increase in PROs within the intervention group compared to controls (p<0.001). This suggests that participants receiving I-PRF report significant improvements in their overall well-being and treatment satisfaction (Table 4).

**Table 4:** Patient-reported outcomes

Time point	Intervention group (n=34)	Control group (n=34)	P-value
Baseline	55.2±10.1	54.8±10.3	0.82
6 weeks post intervention	78.3±8.9	55.1±10.0	<0.001
12 weeks post intervention	82.7±7.5	54.6±9.8	<0.001
24 weeks post intervention	86.2±6.8	54.2±9.5	<0.001

## Discussion

The present study aimed to investigate the efficacy of I-PRF in alleviating TMJ pain. The results from this prospective, randomized, double-blind, placebo-controlled clinical trial demonstrated notable improvements in TMJ pain intensity, jaw function, and PROs for quality of life and satisfaction.

The reduction in TMJ pain intensity observed in the intervention group, as measured by the VAS, is consistent with previous studies that have explored the analgesic effects of PRF in various medical and dental applications [12]. The significant decrease in pain scores at multiple time points, starting from six weeks post intervention and continuing throughout the 24-week follow-up, suggests that I-PRF may offer sustained pain relief for individuals with TMJ pain. This finding aligns with the known regenerative and anti-inflammatory properties of platelet-rich products (PRP), which are thought to modulate the local tissue environment and promote healing [13]. The observed improvement in pain intensity may be attributed to the ability of I-PRF to release growth factors and cytokines, fostering tissue repair and mitigating inflammation in the TMJ region [14]. In the management of TMJ disorders, PRP administration has gained popularity due to its palliative and anti-inflammatory effects [15]. Clinical and radiological comparisons have shown that intra-articular PRP injections decreased TMJ palpation pain more effectively compared with other treatment modalities [16]. Moreover, PRP has been compared with arthrocentesis to evaluate its effectiveness in relieving symptoms of refractory TMJ pain dysfunction syndrome, showing promising results [17]. Overall, the observed reduction in TMJ pain intensity following the intervention with PRF aligns with the known regenerative and anti-inflammatory properties of PRF, which are thought to modulate the local tissue environment and promote healing. The sustained pain relief observed in the intervention group may be attributed to the ability of PRF to release growth factors and cytokines, fostering tissue repair and mitigating inflammation in the TMJ region.

The significant improvement in jaw function, as evidenced by increased maximal mouth opening and enhanced lateral excursions, is a crucial outcome supporting the potential clinical benefits of I-PRF. Similar positive effects on jaw function have been reported in studies investigating PRF in the context of oral and maxillofacial surgery [8,12]. The improvement observed in the intervention group suggests that I-PRF may positively impact the functional aspects of the TMJ, contributing to increased mobility and flexibility. The mechanisms underlying these functional improvements may be multifactorial. PRF's role in tissue regeneration and repair could enhance the structural integrity of the TMJ, leading to improved joint movement and function [18,19]. Additionally, the anti-inflammatory properties of PRF may contribute to reduced joint swelling and improved jaw mobility. In contrast to the results of our study, a systematic review and network meta-analysis of randomized controlled trials suggested that intra-articular injections of PRP had no significant effect on improving TMJ pain and functional outcomes compared with placebo injections [20].

The multifaceted benefits of I-PRF extend beyond pain reduction, as evidenced by significant improvements in quality of life and satisfaction scores in the intervention group. Similar to our results, Sağlam et al. found a significant increase in satisfaction scores in the I-PRF group, highlighting its positive impact on patient satisfaction and well-being [21]. Furthermore, the study by Krishnegowda et al. revealed a significant improvement in patient satisfaction scores with the use of autologous I-PRF in the treatment of atrophic acne scars, emphasizing its positive impact on patient well-being and satisfaction [22]. In the present study, the significant improvements in quality of life and satisfaction scores in the intervention group are indicative of the broader impact of I-PRF beyond pain reduction, highlighting the multifaceted benefits of this intervention in enhancing the overall well-being and satisfaction of individuals with TMJ pain. The satisfaction scores further support the acceptability and perceived efficacy of I-PRF as a therapeutic intervention for TMJ pain. The assessment of PROs through validated questionnaires has allowed for a comprehensive understanding of the subjective experiences of individuals with TMJ pain, providing valuable insights into the impact of interventions such as I-PRF on quality of life, satisfaction, and overall health-related quality of life.

The strength of this study lies in its rigorous and well-defined methodology, incorporating key elements of high-quality clinical research. The adoption of a prospective, randomized, double-blind, placebo-controlled design enhances the internal validity of the study by minimizing selection bias and confounding factors and ensuring that both participants and investigators are blinded to treatment assignments. The primary and secondary outcome measures, particularly the utilization of the VAS for TMJ pain intensity and validated questionnaires for PROs, provide a well-rounded assessment of the intervention's impact. The careful consideration of ethical principles, including obtaining informed consent and obtaining approval from the relevant ethics committee/IRB, underscores the study's commitment to participant welfare and adherence to ethical standards.

Future research in I-PRF for TMJ pain should prioritize large-scale trials with diverse populations, extending follow-up periods for long-term efficacy assessments. Incorporating advanced imaging methods like magnetic resonance imaging (MRI) or cone-beam computed tomography (CBCT) could offer objective structural insights. Optimization of dosages, injection techniques, and frequency, along with comparative studies against existing therapies, will refine treatment protocols. Collaborations between dental and medical researchers may unveil systemic effects, expanding the scope of I-PRF applications in TMJ care. These efforts collectively aim to solidify I-PRF's role, guiding its integration into clinical practice and contributing to the evolution of regenerative approaches for TMJ disorders.

## Conclusions

This study provides compelling evidence supporting the efficacy of I-PRF in mitigating TMJ pain. The significant improvements in pain intensity, jaw function, and PROs underscore the potential clinical utility of I-PRF in the management of TMJ disorders. These findings contribute to the growing body of literature supporting the use of PRP in regenerative medicine and open avenues for further research into optimizing treatment protocols and exploring long-term outcomes.
